# Prospecting *in silico* antibacterial activity of a peptide from trypsin inhibitor isolated from tamarind seed

**DOI:** 10.1080/14756366.2022.2134997

**Published:** 2022-10-28

**Authors:** Gerciane Silva de Oliveira, Amanda Maria de Souza Nascimento, Anna Beatriz Santana Luz, Ana Júlia Felipe Camelo Aguiar, Mayara Santa Rosa Lima, Lídia Leonize Rodrigues Matias, Isabel Rodríguez Amado, Thais Souza Passos, Karla Suzane Florentino da Silva Chaves Damasceno, Norberto de Kássio Vieira Monteiro, Susana Margarida Gomes Moreira, Lorenzo Pastrana, Ana Heloneida de Araújo Morais

**Affiliations:** a Postgraduate Program in Nutrition, Health Sciences Center, Federal University of Rio Grande do Norte, Natal, Brazil; b Postgraduate Program in Biochemistry and Molecular Biology, Center for Biosciences, Federal University of Rio Grande do Norte, Natal, Brazil; c International Iberian Nanotechnology Laboratory, Braga, Portugal; d Nutrition Department, University Center of Rio Grande do Norte, Natal, Brazil; e Analytical Chemistry and Physical Chemistry Department, Science Center, Federal University of Ceará, Fortaleza, Brazil; f The Doctoral Program of Northeast Network in Biotechnology (RENORBIO), Natal, Brazil

**Keywords:** Protease inhibitor, antimicrobial peptides, molecular dynamics simulation

## Abstract

Bacterial infections have become a global concern, stimulating the growing demand for natural and biologically safe therapeutic agents with antibacterial action. This study was evaluated the genotoxicity of the trypsin inhibitor isolated from tamarind seeds (TTI) and the antibacterial effect of TTI theoric model, number 56, and conformation number 287 (TTIp 56/287) and derived peptides *in silico*. TTI (0.3 and 0.6 mg.mL^−1^) did not cause genotoxicity in cells (*p* > 0.05). *In silico*, a greater interaction of TTIp 56/287 with the Gram-positive membrane (GP) was observed, with an interaction potential energy (IPE) of −1094.97 kcal.mol^−1^. In the TTIp 56/287-GP interaction, the Arginine, Threonine (Thr), and Lysine residues presented lower IPE. In molecular dynamics (MD), Peptidotrychyme59 (TVSQTPIDIPIGLPVR) showed an IPE of −518.08 kcal.mol^−1^ with the membrane of GP bacteria, and the Thr and Arginine residues showed the greater IPE. The results highlight new perspectives on TTI and its derived peptides antibacterial activity.

## Graphical Abstract

**Figure:**
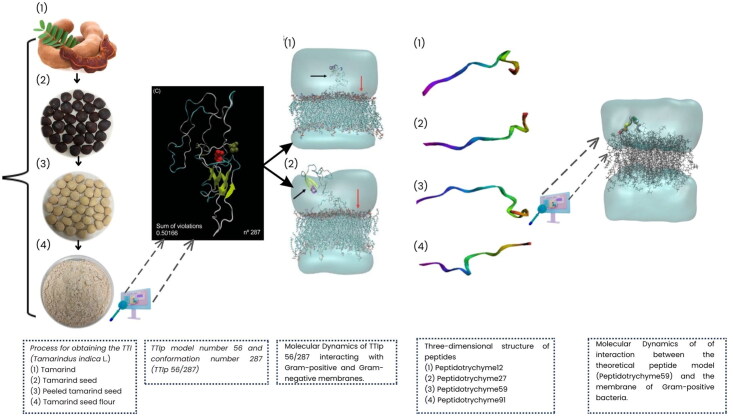

## Introduction

The World Health Organisation (WHO) states that infectious diseases (IDs) can be described in three ways: diseases that cause high mortality rates; diseases that place burdens of disability on populations; and fast-spreading diseases that can have serious global repercussions^1^. Therefore, they are highlighted as the top ten diseases with the highest mortality rates in the world^2^, and no improvement in mortality rates is expected until 2030^3^.

When the ID originates from bacteria, it is recognised as a bacterial infection. These bacterial pathogens contribute to the incidence of diseases transmitted by water or food^4^, and it is estimated that the contamination of these elements can affect 48 million people. Among these, 128,000 need to be hospitalised, and 3000 die annually, according to data from 2020 from the Centres for Disease Control and Prevention (CDC)^5^.

Bacterial antibiotic resistance occurs when bacteria develop the ability to resist the effect of antibiotics intended to inhibit their growth or destroy them^6^. The set of chemicals capable of fighting IDs is known as antimicrobial agents, which effectively prevent, limit, and eliminate the growth of microorganisms. Most of them are obtained from natural sources^7^. Antimicrobial agents exert their mechanisms of action in different ways, i.e. interfering with cell wall synthesis, altering the permeability of the cytoplasmic membrane, promoting changes in protein synthesis, inhibiting nucleic acid synthesis, and interfering with chromosomal replication. The bacterial membrane is an important target for many antibacterial formulations^8^.

In 1998, the WHO estimated that about 60–80% of the population in developing countries needed to use natural therapeutics to prevent or treat diseases^9^. Thus, natural agents with antibacterial activity currently represent a prominent subject^10^. Protease inhibitors (PIs) appear to be promising molecules with antimicrobial activity due to their defence mechanisms against pathogens^10^. They have been identified as excellent candidates for developing new antimicrobial agents in this context. Among PI’s different classes, trypsin inhibitors and/or their derivative peptides have shown antibacterial activities, evidenced in the literature by their bacteriostatic and bactericidal activity^9^.

Prospecting for peptides with antimicrobial activity is a promising strategy. Peptides are protein fragments containing amino acid residues obtained through the action of specific enzymes, by physiological digestion, or enzymatic hydrolysis. They can be reproduced by techniques or methods *in silico*, *in vitro*, or *in vivo*^7^^,^^10^.

Since biomedical applications are intended for these peptides, it is essential to assess their biological safety^11^. Among the tests required by regulatory agencies, such as the Food and Drug Administration (FDA) and the National Health Surveillance Agency (ANVISA) are the genotoxicity tests, which assess whether a substance is toxic to the genetic material^12^^,^^13^. Thus, several standardised tests, due to their excellent reproducibility and reliability, allow for validating the safety of substances for use in humans^13^.

The trypsin inhibitor isolated from tamarind seeds (*Tamarindo indica* L.) (TTI) is a promising protein since it has been studied about its applicability by researchers from the research group Nutrition and Bioactive Substances applied to Health (NutriSBioativoS) at the Universidade Federal University of Rio Grande do Norte (UFRN) in Brazil. Previous studies showed that both TTI, partially or fully purified, showed lower plasma concentrations and relative expression of TNF-α mRNA in a preclinical model. Besides, it promoted the reduction of inflammatory infiltrates in perirenal adipose tissue in Wistar rats with metabolic disorders, denoting its anti-inflammatory potential^14–18^. Thus, considering the multifunctionality of the trypsin inhibitors from tamarind seeds, they are excellent candidates to evaluate their antimicrobial potential, being able to behave as a PI with antimicrobial activity.

Furthermore, the purified TTI molecule was sequenced, totalling 184 amino acid residues. Its structural conformation was modelled, exhibiting the most stable three-dimensional molecular arrangement, model number 56, and conformation number 287 (TTIp 56/287)^19^^,^^20^. Bioinformatics studies are essential tools for theoretical simulation in different processes and analyses. They aim to reduce the number of *in vitro* analyses as they have lower reagent costs and are less time^21^. In this context, this study evaluated the genotoxicity of TTI and the antibacterial effect of the theoretical model of TTI (TTIp 56/287) and its derived peptides, obtained from cleavage *in silico* using combined intestinal enzymes (Trypsin and Chymotrypsin), regarding the antibacterial potential.

## Materials and methods

### Reagents

All the reagents used were analytical grade and obtained from Sigma^®^ (St. Louis, MO) and VETEC Química Fina Ltda^®^ (Rio de Janeiro, Brazil).

### Cellular culture

Chinese hamster ovary cells (CHO-K1, ATCC® CCL-61^™^) were used to evaluate the cytotoxicity and genotoxicity of the TTI sample. Cells were maintained in a basal medium containing Dulbecco’s Modified Eagle Medium (DMEM, Gibco) supplemented with antibiotics (10,000 U mL^−1^ penicillin G and 25 μg.mL^−1^ streptomycin, Gibco), foetal bovine serum (FBS 10%, Gibco) and conditioned in an incubator (PHCbi, model MCO-170AIC-PE) at 37 °C with humidity and with 5% CO_2_. Cells were subcultured when they reached 80 − 90% confluence. All handling occurred in a biological safety cabinet (Pachane laminar flow).

### Obtaining trypsin inhibitor isolated from tamarind seed

Tamarind seeds were obtained from the local market in the city of Natal-RN. They were botanically identified by the Brazilian Institute for the Environment and Renewable Natural Resources (IBAMA) Natal/RN (Brazil). In addition, the material was registered in the National System for the Management of Genetic Heritage and Associated Traditional Knowledge (SisGen) under the number AF6CE9C.

Obtaining the TTI followed the methodology described by Carvalho et al. (14). The seeds were peeled and then grounded in a refrigerated grinder (6 °C) until obtaining fine-grained flour (40 Mesh). Subsequently, 50 mM Tris-HCl buffer, pH 7.5, was added in a proportion of 1:10 (w/v) under constant stirring for 3 h at room temperature. After stirring, it was centrifuged at 10,000 rpm for 30 min at 4 °C to obtain the supernatant identified as crude extract (CE).

The protein fractionation of CE was performed by precipitation with ammonium sulphate at 0–30% and 30–60%, called fraction 1 (F1) and fraction 2 (F2), respectively. After each precipitation step, each saturation strip was centrifuged under the same reported conditions. Each precipitate obtained was resuspended in Tris–HCl buffer (50 mM, pH 7.5) and dialysed in a membrane (14 kDa) against distilled water for three days, with an exchange every 24 h and finally against the same extraction buffer, for 2 d, being replaced every 24 h.

The fraction with the highest antitrypsin activity (F2) was applied to a Trypsin-Sepharose affinity column (CNBr-activated Sepharose^™^ 4B, GE Healthcare, Chicago, IL, USA), pre-equilibrated with Tris–HCl (50 mM, pH 7.5). Proteins not retained on the column were eluted with the same extraction buffer. Proteins retained on the matrix were eluted with HCl (5 mM), collected in 5 mL aliquots, and then treated as the eluate. This eluate was dialysed against extraction buffer for 2 d, with buffer replacement every 24 h, and later lyophilised, identified as TTI, and stored at −20 °C for the other procedures.

The degree of purity and molecular weight of the isolated protein were verified by electrophoresis in discontinuous and denaturing polyacrylamide gel (SDS-PAGE), according to Laemmli^21^. To estimate the molecular weight of the isolated protein, the molecular weight marker Fisher BioReagents EZ-Run Rec Protein Ladder (Thermo scientific^TM^, Waltham, MA) was used with molecular weights of 200, 150, 120, 100, 85, 70, 60, 50, 40, 30, 15, and 10 kDa. Protein quantification was determined by the Bradford method^22^ using bovine serum albumin (BSA) as a standard.

### Cytotoxicity profile

To determine the effects of TTI on cell viability, an assay was performed with the reagent 3–(4,5-dimethylthiazol-2-yl)-2,5-diphenyltetrazolium bromide (MTT). It is a colorimetric test used to assess cell viability^23^, which follows the rules established by the international standard [ISO 10993–5 – Biological evaluation of medical devices]. The CHO-K1 cells were seeded in a 96-well plate (5 × 10^3^ cells.well^–^^1^) and incubated for 16 h in a controlled atmosphere, 5% CO_2_ at 37 °C. For the negative control, cells maintained in the basal medium were used. The TTI concentrations tested (0.3 and 0.6 mg.mL^−1^) were based on the study by Costa et al.^24^

At the end of 24 h, the medium was removed, and 100 µL of MTT solution (1 mg.mL^−1^ in PBS) was added to each well. After 4 h of incubation at 37 °C, the supernatant was removed, and the insoluble crystals of formazan (precipitate) produced by metabolically active cells were suspended and dissolved with 100 μL of DMSO. The solution formed absorbs in the visible region, thus allowing the quantification by spectrophotometry. Absorbance was measured at 570 nm using a microplate reader (BioTek Instruments, Winooski, VT, USA)^25^. Three independent experiments and triplicate.

(1) $$\text{MTT reduction }\left(\%\right)=\frac{{\text{A}}_{\text{sample}}{}_{\left(570\;\text{nm}\right)}\times 100}{{\text{A}}_{\text{control}\;(570\;\text{nm})}}\;$$

The percentage of cell proliferation (%) was expressed according to Equation (1), in which A_control_ is the absorbance of the negative control and A_sample_ is the absorbance of the samples measured at 570 nm.

### Genotoxicity profile

The genotoxicity potential was determined by the cytokinesis-blocked micronucleus assay (CBMN). The CBMN assay was performed using CHO-K1 cells, which is an established cell line for CBMN assays, following the guidelines for the *in vitro* mammalian cell micronucleus Test N^o^. 487: *In vitro* mammalian cell micronucleus test (OECD, 2010)^26^ and developed according to the Fenech protocol^27^. Cells were seeded in a 24-well plate (2 × 10^4^ cells.well^−1^) and cultured in 500 μL of basal medium for 24 h under controlled conditions at 37 °C, in 5% CO_2_.

Cells were grown to 80% confluence, and the medium was replaced by a fresh medium containing TTI (0.3 and 0.6 mg.mL^−1^), and cells were incubated for 24 h. Then, the medium was aspirated, and the cells were washed with PBS. Subsequently, a fresh medium supplemented with cytochalasin B (Cyt B; Sigma^®^, St. Louis, MO, USA) was added, and the cells were incubated for another 24 h. Afterward, the cells were washed with PBS, detached using 200 μL of 0.025% Trypsin (0.05% EDTA) for 5 min, and suspended in a culture medium. After centrifuging, the cells were suspended in fixation buffer at 4 °C, consisting of methanol and glacial acetic acid (9: 1 *v/v*). Washing in fixation buffer was repeated three times.

The cells were placed on the slides. Then the slides were dried and stained with a 4% aqueous solution of Giemsa for 5 min. Finally, the cells were analysed under an optical microscope (OLYMPUS CX22LED) with 40× objectives, where 3000 binucleated cells per treatment in each experiment were analysed for the formation of micronuclei (MN), nucleoplasmic bridges (NPBs), and nuclear buds (NBUDs), as proposed by Fenech^27^. Three independent experiments were performed, and the results were expressed as the number of occurrences of alterations per 1000 binucleated cells.

The cell division index (CDI) measures the proliferative potential of viable cells, indicating the cytostatic effect of the material tested. The CDI of each treatment was calculated, and the treatment with mitomycin C (0.1 μg.mL^−1^) (added simultaneously with cyt-B) for 24 h was considered the positive control. Cells cultured in a basal medium were used as a negative control.

Finally, the following calculation was performed:

(2) $$\text{CDI}\;=\frac{\left(M1\;+\;2M2\;+\;3M3\;+\;4M4\right)}{N}$$

*M*1–*M*4 represent the number of cells with 1–4 nuclei, respectively, and *N* is the total number of labelled viable cells. The lowest CDI value is 1, determined when all viable cells have not divided during cytokinesis blockade. A CDI value of 2 indicates that all viable cells have completed a nuclear division and are binucleated.

### In vitro gastrointestinal digestion simulation

The digestion pattern and enzymatic susceptibility of TTI were determined using the simulated *in vitro* digestion assay conducted according to the INFOGEST protocol, updated and described according to Brodkorb et al.^28^

TTI was evaluated at a concentration of 56 mg mL^−1^. Water was used as a negative control using the same initial volume (1 mL), according to Matias et al.^29^ During the test, in the three phases of digestion, both TTI and water were kept under the same conditions: temperature of 37 °C (simulating body temperature), and mechanical agitation at 200 rpm.

The simulated digestion took place in three sequential phases: oral, gastric, and intestinal, and in the last two phases, aliquots were collected at 20, 60, and 120 min for digestion monitoring analyses. The digestion process was monitored by tricine polyacrylamide gel electrophoresis at different times of the gastric and intestinal phases, and the antitrypsin activity was evaluated at the end of these phases.

#### Oral phase

For the initial step of *in vitro* digestion, 56 mg of TTI was diluted in 1 mL of Milli-Q water, and 800 μL of Salivar Simulator Fluid (SSF) was added; 5 μL of calcium dichloride (0.3 M) and 195 μL of Milli-Q water, to have a final volume equal to 2 mL. Then, the mixture was shaken in a Thermomixer (Eppendorf, Hamburg, Germany) for 2 min at 200 rpm and 37 °C. At the end of this phase, the final concentration of TTI was 28 mg.mL^−1^.

#### Gastric phase

At the end of the oral phase, 1,600 μL of Gastric Simulator Fluid (GSF) was added to the TTI mixture; 100 μL of bovine pepsin solution (21.12 mg.mL^−1^ in water); 1.0 µL of calcium chloride (0.3 M); 233 μL Milli-Q water and 66 μL hydrochloric acid (1 M), required to adjust the pH to 3.0. For the negative control (water), 11 μL of hydrochloric acid (1 M) were needed to reach pH 3.0 and Milli-Q water. TTI and water were incubated in this phase at the same temperature and agitation conditions as in the oral phase for 120 min. The final volume of the solution in this phase was 4 mL, being TTI at a concentration of 14 mg.mL^−1^.

### Intestinal phase

After 120 min, the gastric phase was stopped by the addition of 1190 μL of Intestinal Simulated Fluid (ISF), 700 μL of Pancreatin (82,432 mg.mL^−1^ of ISF), 350 μL of a solution containing bile salts (82,902 mg.mL^−1^ of ISF), and 5.6 μL of calcium chloride (0.3 M). Furthermore, after adding these solutions, the pH was adjusted to 7. The volume was completed up to a volume of 8 mL. Then, this mixture was maintained under the same stirring conditions, temperature, and time described as in the gastric phase. The final TTI concentration was 7 mg.mL^−1^.

Digestion was stopped by cooling the samples in an ice-water bath. Then, samples were submitted to refrigerated centrifugation for 10 min at 3000 rpm. Then, supernatants from digested samples were frozen at −80 °C until further analysis.

#### Monitoring of digestion

The digestion process was monitored using 16.5% Tricine Mini-PROTEAN^®^ TGX^™^ Precast Protein Gels, 12-well (Bio-Rad, Hercules, CA, USA) gel electrophoresis^30^.

Aliquots from each phase (oral, gastric, and intestinal) were diluted in sample buffer (10x Tris/Tricine/SDS Running Buffer-BIO-RAD) and vortexed. Then, they were heated (Eppendorf ThermoMixer C) at 95 °C for 5 min and applied to the gel. The gel running condition was at 65 mA for approximately 100 min. After running, the gel was fixed in a fixing solution of methanol, acetic acid, and water (4:1:5 *v/v/v*). Then, the gel was stained in Coomassie Brilliant Blue G-250 solution (Bio-Rad). The standard low molecular weight marker, the PageRuler Unstained Low Range Protein Ladder (100, 30, 25, 20, 15, 10, 5, and 3.4 kDa) (Thermo scientific^™^), was used to monitor the digestion products used.

The evaluation of trypsin inhibitory activity was also performed after simulated *in vitro* digestion of TTI. It was tested according to the protocol of Kakade et al.^31^, using Nbenzoyl-DL-arginine-p-nitroanilide (BApNa/1.25 Mm) as substrate. They were collected and analysed at the final time (120 min) of the gastric and intestinal phases.

### In silico prospection for TTIi (TTI 56/287) and its derivative peptides with antibacterial potential

#### In silico proteolytic cleavage of TTIp 56/287

For *in silico* cleavage, the entire sequence of purified TTI was used [model number 56, in conformation number 287 (TTIp 56/287)] previously predicted by de Medeiros et al.^19^ Thus, TTIp 56/287 was subjected to a theoretical cleavage, using the PeptideCutter analysis tool obtained from the ExPASy server^32^. In this cleavage, considering the results obtained with the *in vitro* simulation of the digestion test, enzymes (Trypsin and Chymotrypsin/replacing Pancreatin) were combined.

#### Selection of peptides for analysis by molecular dynamics (MD)

The peptides obtained from the theoretical cleavage were selected from the alignment with TTIp 56/287 using the CLUSTAL W server^33^. Subsequently, identifying the amino acids in these peptides was performed, considering their respective positions according to the alignment performed.

Therefore, peptides were selected for MD analysis that presented the following characteristics: a sequence greater than eight amino acids. Additionally, for sequences with eight or nine amino acids, Glycine (Gly) molecules were added to the peptides to obtain their respective three-dimensional structures; (2) the amino acids that most interacted with the bacterial lipid bilayer that presented the lowest interaction potential energy (IPE), according to the MD simulation, proposed in this study for TTIp 56/287.

The three-dimensional structures of the potential peptides were obtained using the trRosetta server^34^. The theoretical models were validated using the MolProbity server by analysing essential parameters, such as the Ramachandran graph, bond sizes, and angles^35^.

The prediction of the cationic amphipathic helix structure of each peptide was also performed using the HeliQuest server, which provides information, such as hydrophobicity, hydrophobic moment, and net charge at pH 7^36^. The Peptide property calculator (PepCalc) calculated the hydrophobicity characteristics. The peptide with the most stable model and structure, that is, with the lowest values of residues with bad bonds and angles and better values of favoured Ramachandran and MolProbity score, as well as greater hydrophobicity, hydrophobic moment, net charge, and a polar amino acid sequence, associated characteristics mentioned above, was selected for analysis by MD.

#### Interaction of TTIp 56/287 and its selected peptide with the bacterial membrane by MD

The 3D structure of TTIp 56/287 and the selected peptide validated by MolProbity were used for MD analysis. To simulate the theoretical model of the membrane of Gram-negative (GN) and GP bacteria, the lipid bilayer model generated by the Membrane Builder module using the CHARMM-GUI server (http://www.charmmgui) was applied. org/), in order to match the GN bacterial membrane model^36^, a mixture with a 3:1 molar ratio of 1-Palmitoyl-2-oleoyl-sn-glycero-3-phosphoethanolamine (POPE). was used to 1-Palmitoyl-2-oleoyl-sn-glycero-3-anionic phosphoethanolamine (POPG) and GP^37^ a mixture with a 3:1 molar ratio of POPG to anionic POPE.

#### Molecular dynamics (MD)

MD simulations were performed using the initial three-dimensional structure of TTIp 56/287 and selected peptide. The MD simulation for the selected peptide was performed only with the lipid bilayer of bacteria that showed the lowest IPE. The MD was performed using the GROningen MAChine for Chemical Simulations (GROMACS) package version 2018.4^38^ implemented with the force field Chemistry at Harvard Macromolecular Mechanics 36 (CHARMM36)^39^. Water molecules of the TIP3P90 type were used to solvate the simulated systems, and counter-ions were added to neutralise the system^40^. The long-range interactions were modelled using the Ewald particle mesh sum (PME)^41^ with a cut-off of 1.2 nm, as well as the same calculation was used to evaluate the Van der Waals interactions. Covalent bonds were contained using LINCS^42^. The lipid bilayer used for all membrane simulations was positioned on the XY axis, composed of 120 lipids. For GP 30, there were POPE molecules and 90 POPG molecules, and for GN 90 POPE and 30 POPG molecules. And in the z-axis, a thick layer of water of ∼40 Å was maintained on both sides of the lipid bilayer.

The steepest descent algorithm has minimised the geometry of the TTIp 56/287 systems for 5000 steps with a tolerance of 500 kJ mol^−1^ nm^−1^. Two short 25 ps equilibrium dynamics simulations were performed with the NVT set (number of particles (N), system volume (V), and temperature (T) are constant), followed by four short 25 ps equilibrium dynamics, 100, 100, and 225 ps with the NVT set. Finally, 100 ns MD simulations using NVT were performed for each system to determine the adsorption of TTIp 56/287 with the lipid bilayers. The selected peptide system geometry was optimised through the steepest descent algorithm^43^ with an energy tolerance of 10 kJ mol^−1 ^nm^−1^ through 10,000 steps. Two equilibrium dynamics were performed with a time of 100,000 ps each. The first used the canonical ensemble NVT with the V-rescale method^44^ at a temperature of 310 K. The second equilibrium dynamics was performed with the isothermal-isobaric ensemble through the Parrinelo-Rahman^45^ barostat at a pressure of 1.0 bar. Finally, the production MD was performed for the system with a simulation time of 200 ns, maintaining the temperature of 310 K in ensemble NVT, to determine the adsorption of the selected peptide with the lipid bilayer through IPE.

#### Interaction potential energy (IPE)

The IPE was used to explore the adsorption between TTIp 56/287, the selected peptide, and the lipid bilayer. It defines the total energy of interaction between two groups^46^. For this, the equation below was used.

(3) $$\begin{array}{c} {\text{N}}_{\text{i}}\;\;{\text{N}}_{\text{j}}\\ {\text{IPE}}_{i,j}=\sum \;\sum {\text{V}}_{\text{vdW}}\left(r_{ij}\right)\;+{\text{ V}}_{\text{elec}}\left(r_{ij}\right)\\ \text{i}\;\;\;\text{j}\neq \text{i} \end{array}$$

IPE*_ij_* is the interaction energy between a group of atoms *i* and a group of atoms *j*, in this work, represented by the group of atoms of the system (TTIp 56/287 and selected peptide) – bacterial membrane and the number of atoms of the same peptide; *Ni* and *Nj* are the total numbers of atoms in groups *i* and *j*, respectively. VvdW (*r_ij_*) is the contributions of Van der Waals and Velec (*r_ij_*), the electrostatic contributions. This method is commonly used to define the interaction energies between protein-ligand and protein–protein^47^. It can also quantify the interaction between specific amino acids and the surrounding molecules^48^.

### Statistical analysis

Data were expressed as mean ± standard deviation of the mean (SD) of three independent determinations. Statistical analyses were performed using GraphPad Prism version 9.4.0 (Graphpad Software Inc., La Jolla, CA). Analysis of variance (ANOVA) and Tukey’s post‐test were used for single and multiple comparisons. *p* Values less than 0.05 were considered statistically significant (*p < 0.05; **p < 0.01; ***p < 0.001; and ^****^p < 0.0001).

## Results

### Obtaining trypsin inhibitor isolated from tamarind seed

**Figure 1.**
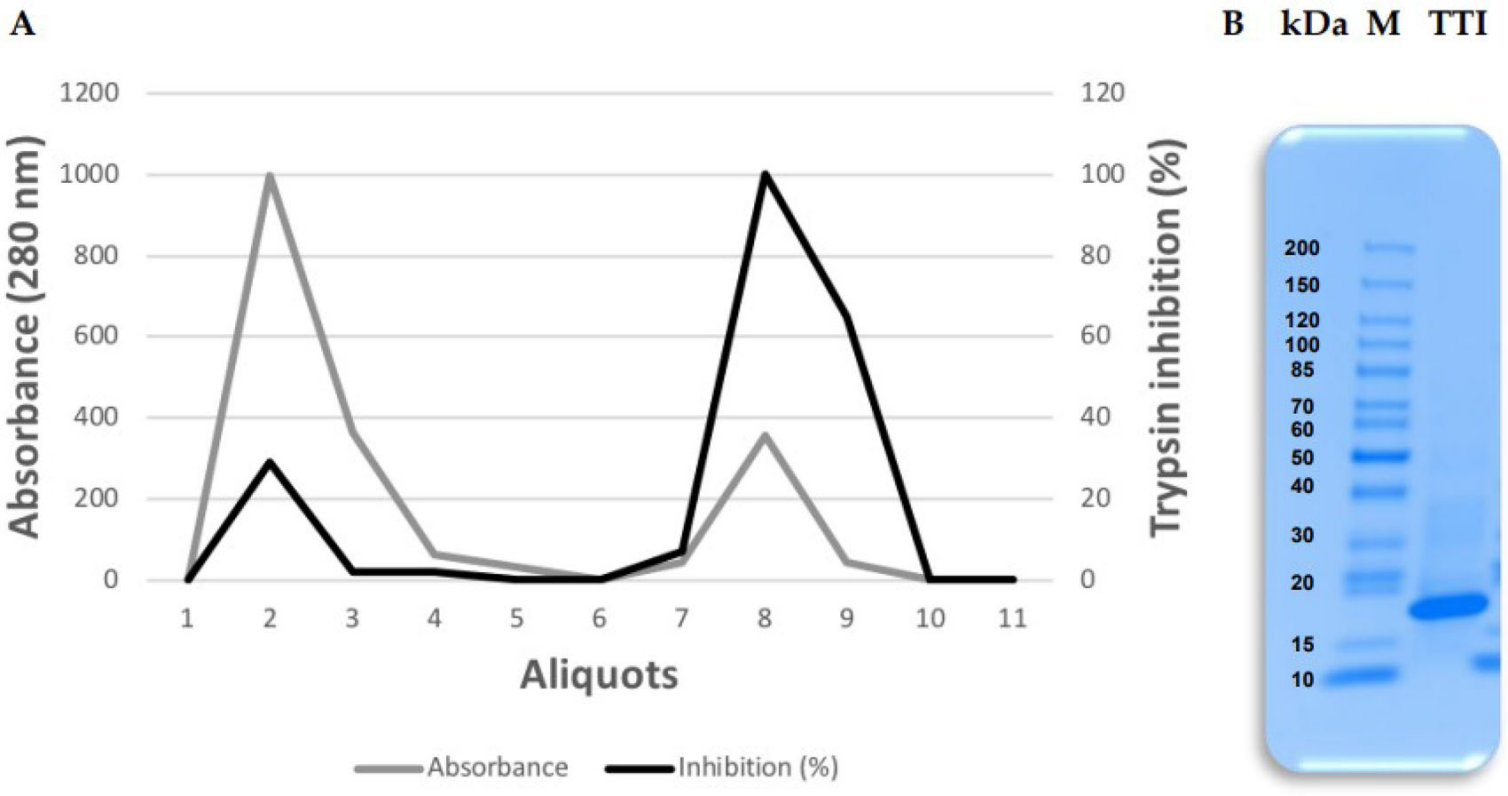
Obtaining the trypsin inhibitor isolated from tamarind seeds (TTI). (A) Chromatographic profile of protein fraction 2 obtained from tamarind (*Tamarindus indica* L.) seed flour extract submitted to Trypsin-Sepharose 4B CNBr affinity chromatography. Column calibration was performed with 50 mM Tris–HCl buffer, pH 7.5, and the retentate was eluted with a 5 mM HCl solution at a flow rate of 0.5 mL.min^−1^. (B) 16.5% Tricine gel electrophoresis stained with Coomassie Blue. M: Tracer (Thermo Scientific^™^); TTI: 15 µg.mL^−1^.

Protein fraction 2 (saturated with 30–60% ammonium sulphate) was submitted to affinity chromatography on Trypsin-Sepharose CNBr 4B to isolate the TTI. The chromatography column was calibrated with 50 mM Tris–HCl buffer, pH 7.5, and the eluate from the proteins adsorbed to the column was collected with the addition of 5 mM HCl at a flow rate of 0.5 mL.min^−1^, presenting 97 0.9% antitrypsin activity (Figure 1(A)). The isolated ITT is observed, which is detected by denaturing sodium dodecyl sulphate-polyacrylamide gel electrophoresis (SDS-PAGE) at 16.5%, being possible to identify a predominant protein band of approximately 20 kDa (Figure 1(B)).

### Assessment of cytotoxicity in of CHO-K1 cells

**Figure 2.**
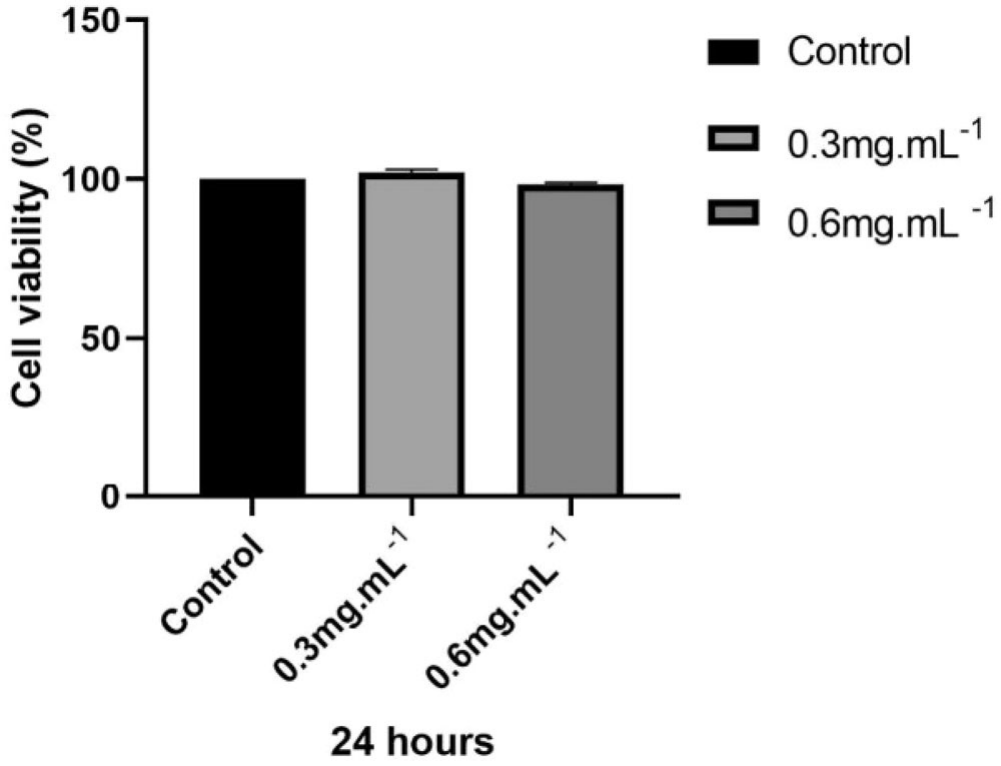
Cell viability of CHO-K1 cells treated with TTI assessed by the MTT assay. NC: negative control (cells in basal medium); TTI 0.3: trypsin inhibitor isolated from tamarind seeds at a concentration of 0.3 mg.mL^−1^; TTI 0.6: trypsin inhibitor isolated from tamarind seeds at a concentration of 0.6 mg.mL^−1^. Results represent the mean ± SD of three independent experiments performed in three replicates. The statistical difference was evaluated according to analysis of variance (ANOVA) and Tukey’s post-test, considering the statistical difference when *p* < 0.05.

TTI cell viability was evaluated by the MTT assay at different concentrations using the CHO-K1 cell line. Results showed all TTI concentrations had similar cell viability values to the negative control and did not differ statistically (Figure 2).

### Assessment of genotoxicity in of CHO-K1 cells

**Figure 3.**
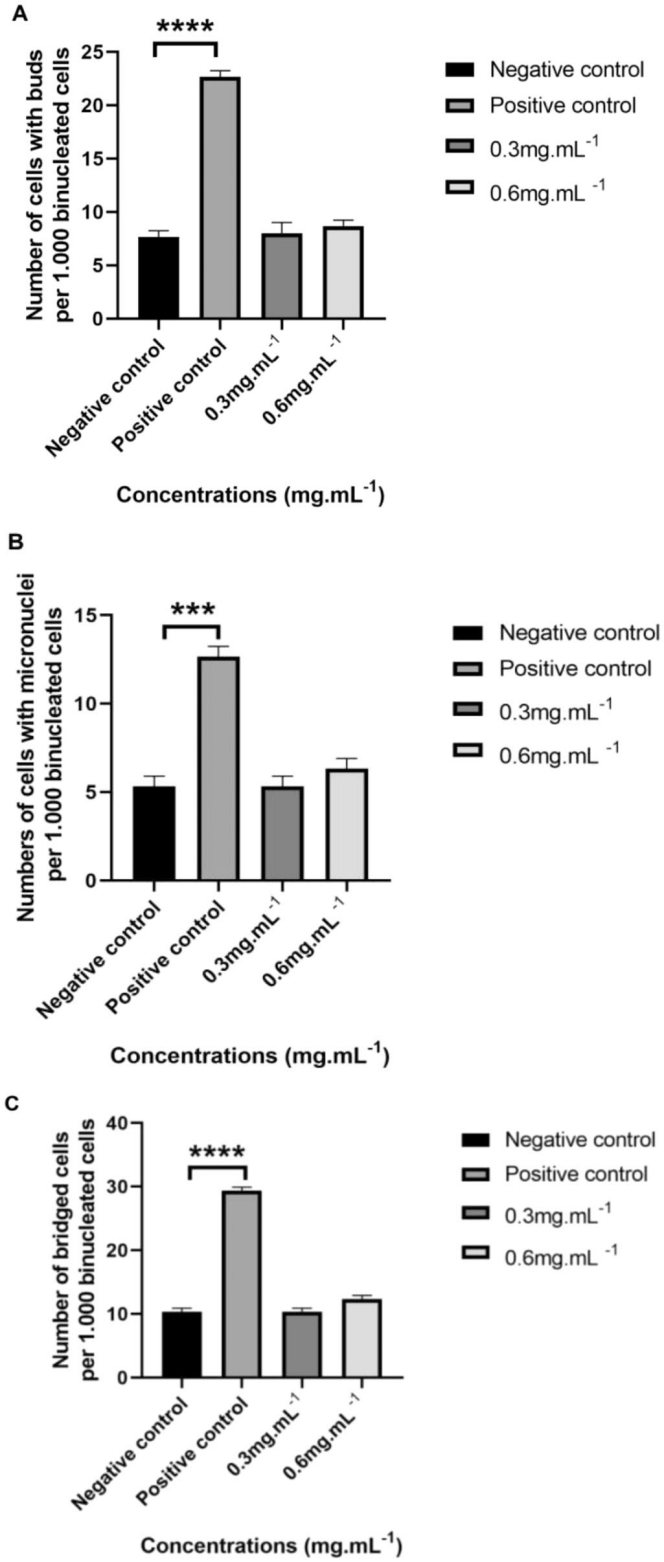
Number of nuclear alterations per 1000 binucleated cells analysed by cytokinesis-blocked micronucleus assay (CBMN) in 24 h cultures of CHO-K1 cells. (A) Nuclear buds. (B) Micronuclei. (C) Nucleoplasmic bridges. CHO-K1 cells: Chinese hamster ovary cells. Cells were cultured in basal medium (NC: negative control) or in the presence of mitomycin C (PC: positive control). Results represent the mean ± SD of two independent experiments. (*p ≤ 0.05; **p ≤ 0.01; ***p ≤ 0.001; ^****^p ≤ 0.0001 statistically significant in relation to NC according to ANOVA and Tukey’s post-test).

The CBMN assay was performed using CHO-K1 cells to assess whether TTI induced nuclear alteration at different concentrations (0.3 mg.mL^−1^ and 0.6 mg.mL^−1^). Based on the results, no genotoxicity was observed. A statistically significant difference was not observed between the cells’ exposure to TTI and the negative control (cells in basal medium). However, the positive control (cells in the presence of mitomycin C) differed statistically from the negative control in all nuclear alterations evaluated in the binucleated cells: the presence of NPBs and NBUDs (^****^p ≤ 0.0001) and MN (***p ≤ 0.001) (Figure 3).

### Simulation of in vitro gastrointestinal digestion of TTI

#### Monitoring of antitrypsin activity

The *in vitro* digestion of TTI, simulating the gastric and intestinal phases after 20, 60, and 120 min, was evaluated for the antitrypsin activity of TTI. Only the digestion products of the gastric phase maintained the inhibitory anti-tryptic activity. This specific activity of the intact TTI confirmed its digestion in the intestinal phase and susceptibility only to the enzymes Trypsin and Chymotrypsin, the proteolytic enzymes contained in Pancreatin (Table 1).

**Table 1.** Percentage of antitrypsin activity of trypsin inhibitor isolated from tamarind seeds (TTI) under *in vitro* simulated gastrointestinal digestion conditions.

Antitrypsin activity (%)			
	Digestion phases		
	No digestion Mean (SD)	Gastric Mean (SD)	Intestinal Mean (SD)
Water	−	0.00 (0.009)	0.00 (0.019)
TTI intact*	96.33 (0.001)	−	−
TTI digested**	−	96.64 (0.001)	0.00 (0.008)

The concentration of TTI (0.7 mg.mL^−1^) used to evaluate the antitrypsin activity was the same in both conditions: *TTI intact without passing through the conditions of simulated digestion; **TTI subjected to *in vitro* simulated digestion conditions. (−) Antitrypsin activity was not performed. The digestion protocol used was the INFOGEST static *in vitro* simulation of gastrointestinal food digestion (Brodkorb et al.^28^). For antitrypsin activity, 100 µL of TTI and Nbenzoyl-DL-arginine-p-nitroanilide (BApNa/1.25 mM) were used as substrate.

### In silico prospection of TTI (TTIp 56/287) and its derivative peptides with antibacterial potential

#### MD simulation

**Figure 4.**
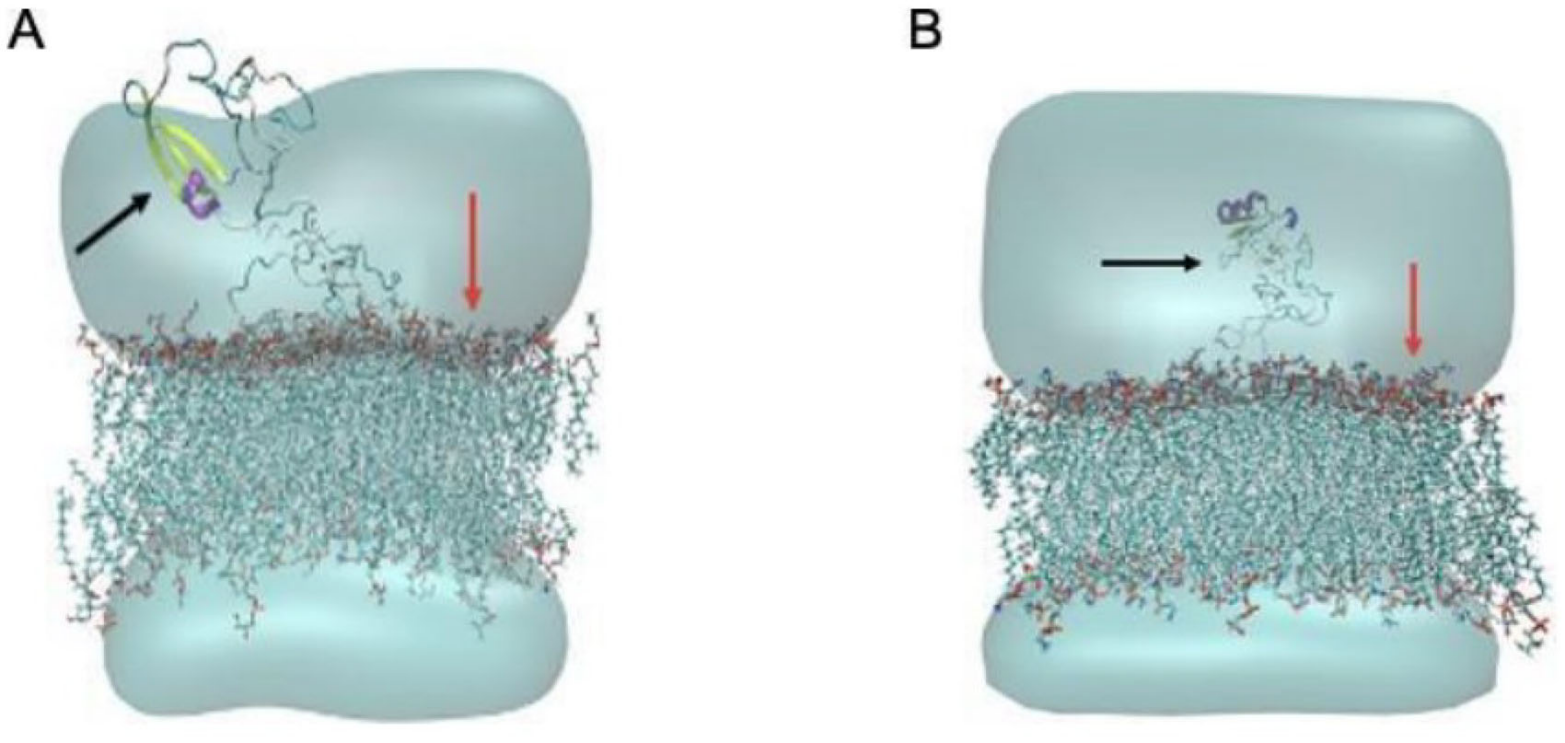
Structural behaviour between model number 56 in conformation number 287 of TTIp (purified trypsin inhibitor from tamarind seeds – TTIp 56/287), with the bilayer model in proportions of 3:1 of 1-palmitoyl-2- oleoyl-sn-glycero-3-phosphoglycerol (POPG) to anionic 1-palmitoyl-2-oleoyl-sn-glycero-3-phosphoethanolamine (POPE) in Gram-positive membrane representation (A), and 1:3 POPG- POPE in Gram-negative membrane representation (B) generated from Molecular Dynamics. The arrows points to the bacterial membrane, and to TTIp 56/287.

In this study, the MD simulation of TTIp 56/287 was performed using the lipid bilayer model through the CHARMM-GUI server with bacterial membranes GP and GN. In the images generated from MD, it is possible to visualise the structural behaviour and interactions of TTIp 56/287 at the atomic level with bacterial membranes, GP (Figure 4(A)), and GN (Figure 4(B)), evidencing its dynamic behaviour.

**Figure 5.**
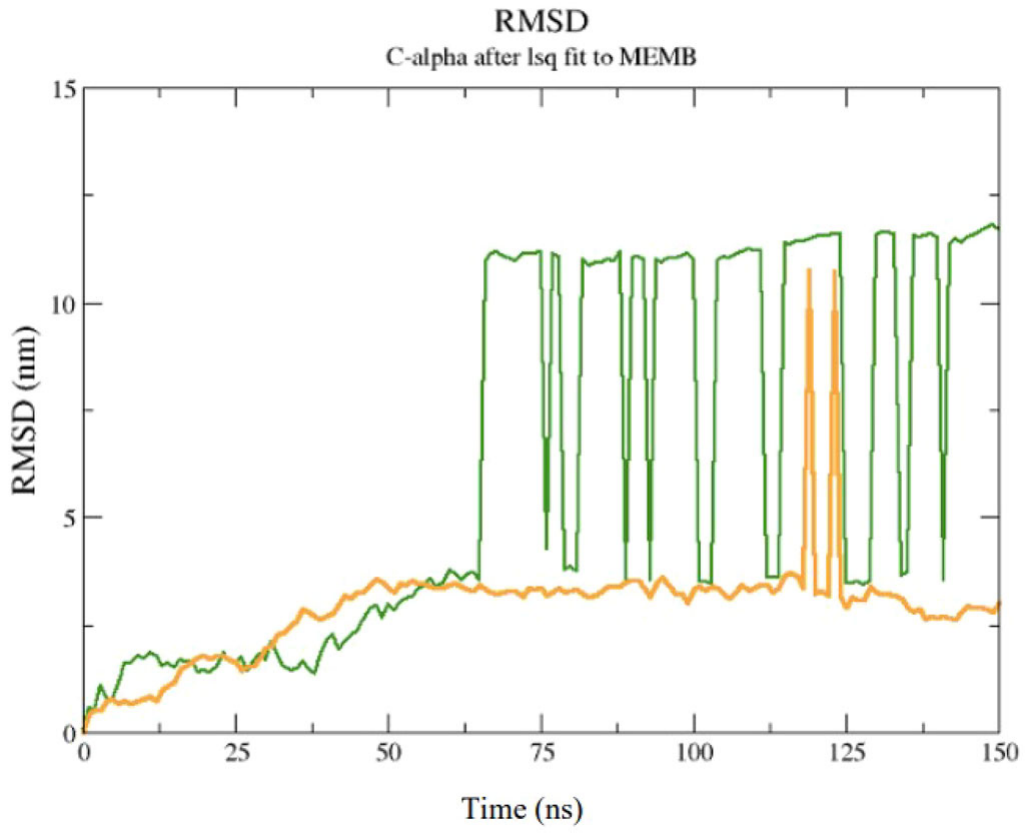
Root mean square deviation (RMSD) graph of molecular dynamics simulation between model number 56, in conformation number 287 of TTIp (purified trypsin inhibitor from tamarind seeds – TTIp 56/287) with the bilayer model, in the 3:1 ratios of 1-palmitoyl-2-oleoyl-sn-glycero-3-phosphoglycerol (POPG) to anionic 1-palmitoyl-2-oleoyl-sn-glycero-3-phosphoethanolamine (POPE) in the Gram-positive (ten peaks), and 1:3 POPG-POPE in Gram-negative membrane representation (two peaks) generated from molecular dynamics.

To evaluate the potential energy of interaction of the TTIp 56/287-bacterial membranes system, the MD root mean square deviation (RMSD) plot was constructed. This graph reveals the value of the mean square deviation of the atoms of the TTIp 56/287 systems with the GP bacterial membrane (represented in green) and TTIp 56/287 with the GN bacterial membrane (represented in yellow) as a function of time in an aqueous system, and consequently the interaction and movement between its atoms. Taking the time interval between the minimum and maximum points at which the equilibrium of these systems occurs, the IPE is calculated (Figure 5).

It is possible to observe that the equilibrium of the TTIp 56/287–GP system occurred after 50 ns, while for the TTIp 56/287–GN system, it occurred after 100 ns. Therefore, the energy value was calculated, including these intervals (Tables 2 and 3).

**Table 2.** Interaction energy of the molecular dynamics simulation between model number 56, in conformation number 287 of TTIp (purified trypsin inhibitor from tamarind seeds – TTIp 56/287), with the bilayer model in proportions of 3:1 from 1-palmitoyl-2-oleoyl-sn-glycero-3-phosphoglycerol (POPG) to anionic 1-palmitoyl-2-oleoyl-sn-glycero-3-phosphoethanolamine (POPE) in Gram-positive membrane (GP) representation in molecular dynamics.

Interaction between TTIp-GP	IPE (kcal.mol^−1^)
Coul-SR: Protein-MEMB	−867.408
LJ-SR: Protein-MEMB	−227.565
Total	−1094.97

**Table 3.** Interaction energy of the molecular dynamics simulation between model number 56, in conformation number 287 of TTIp (purified trypsin inhibitor from tamarind seeds – TTIp 56/287), with the bilayer model in proportions of 1:3 from 1-palmitoyl-2-oleoyl-sn-glycero-3-phosphoglycerol (POPG) to anionic 1-palmitoyl-2-oleoyl-sn-glycero-3-phosphoethanolamine (POPE) in Gram-negative membrane (GN) representation from molecular dynamics.

Interaction between TTIp-GN	IPE (kcal.mol^–1^)
Coul-SR: Protein-MEMB	–348.538
LJ-SR: Protein-MEMB	–95.8026
Total	−444.337

The potential energy of interaction of the TTIp 56/287-GP system was the most negative, so it is possible to highlight that this interaction is more potent. The present analysis elucidates the information atomistically for the level of interaction between TTIp 56/287 and two different models of bacterial membranes, with more significant interaction observed with GP membranes.

**Figure 6.**
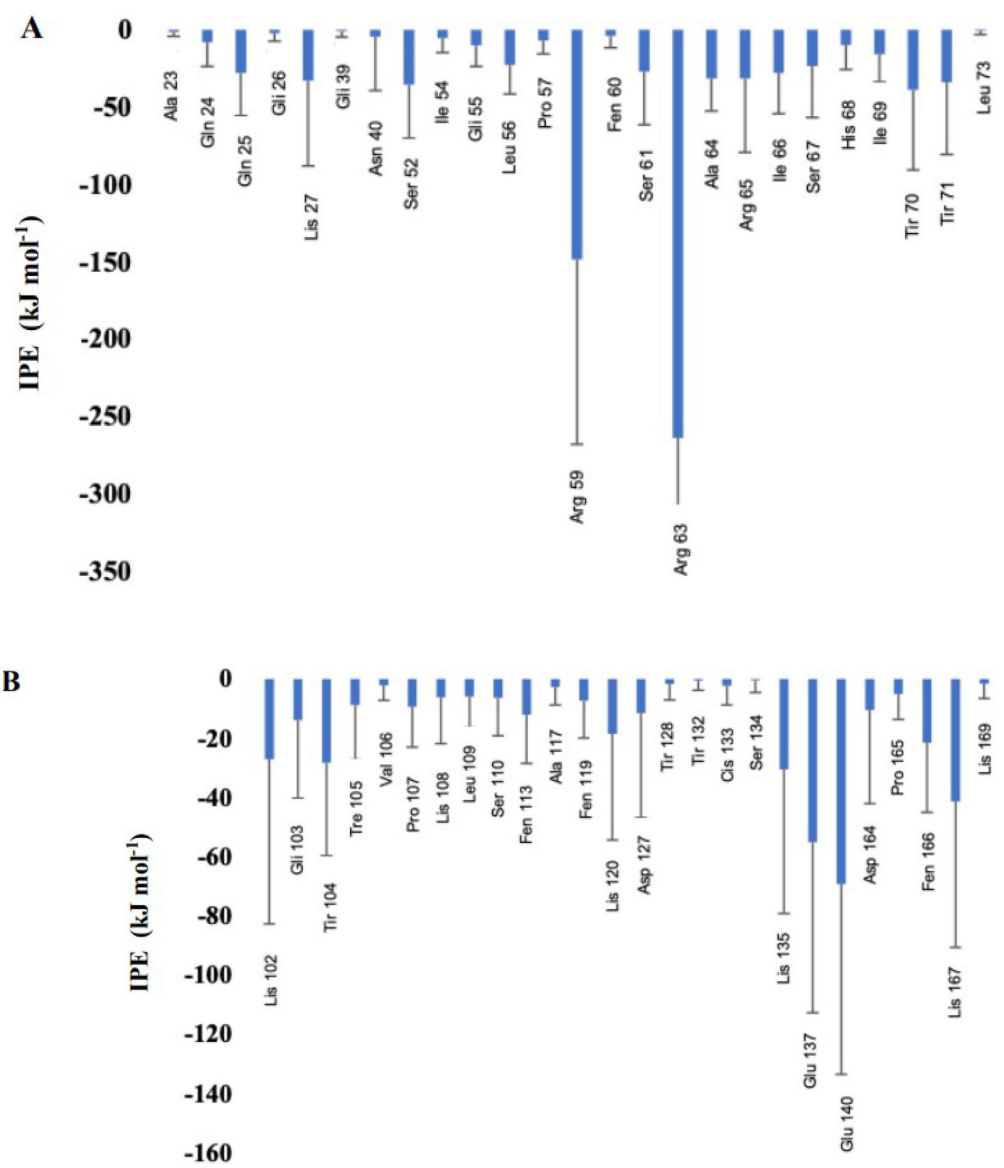
Interaction potential energy (IPE) between amino acids of model number 56, in conformation number 287 of TTIp (purified trypsin inhibitor from tamarind seeds – TTIp 56/287), with the bilayer model in the proportions of 3: 1 of 1-palmitoyl-2-oleoyl-sn-glycero-3-phosphoglycerol (POPG) to anionic 1-palmitoyl-2-oleoyl-sn-glycero-3-phosphoethanolamine (POPE) in Gram-positive membrane representation (A), and 1:3 POPG-POPE in Gram-negative membrane representation (B) generated from molecular dynamics.

Subsequently, it was observed, through MD, which amino acids present in TTIp 56/287 were at a minimum distance of 5 Â from the membranes to identify which of them had the highest interaction energy (Figure 6(A,B)), highlighting the residues of Arginine, Threonine (Thr), and Lysine amino acids.

#### TTI in silico cleavage (TTIp 56/287)

In the *in silico* prospection of peptides derived from TTI with antibacterial potential, the theoretical cleavage of TTIp 56/287 was performed with the combined enzymes Trypsin and Chymotrypsin, and 52 peptides were obtained (Supplementary Table 1). Among these peptides, six were selected, following the criteria of amino acid sequence size (*n*= >8), and those that presented sequences with eight or nine amino acids Gly molecules were added so that it was possible to obtain their three-dimensional structures (Table 4).

**Table 4.** Primary sequences and characteristics of peptides with more than ten amino acid residues generated by the ExPASy server from the *in silico* hydrolysis of the trypsin inhibitor purified from tamarind seeds [model number 56, conformation number 287 (TTIp 56/287)] with the combined enzymes chymotrypsin and trypsin.

Enzymes involved in hydrolysis	Cleavage site position	Cleavage enzymes name	Resulting peptide sequence	Peptide length [aa]	Peptide mass [Da]
Trypsin and Chymotrypsin	12	Chymotrypsin	DTVHDTDGQVPL	12	1296.356
Trypsin and Chymotrypsin	27	Trypsin	ILPAQQGKGG	10	854.017
Trypsin and Chymotrypsin	43	Chymotrypsin	SNDDDGNCPL	10	1049.034
Trypsin and Chymotrypsin	59	Trypsin	TVSQTPIDIPIGLPVR	16	1706.015
Trypsin and Chymotrypsin	91	Trypsin	TIAPACAPKPAR	12	1195.445
Trypsin and Chymotrypsin	181	Chymotrypsin	VDEESSEEWG	10	1109.068
	Result = 6 peptides				

**Figure 7.**
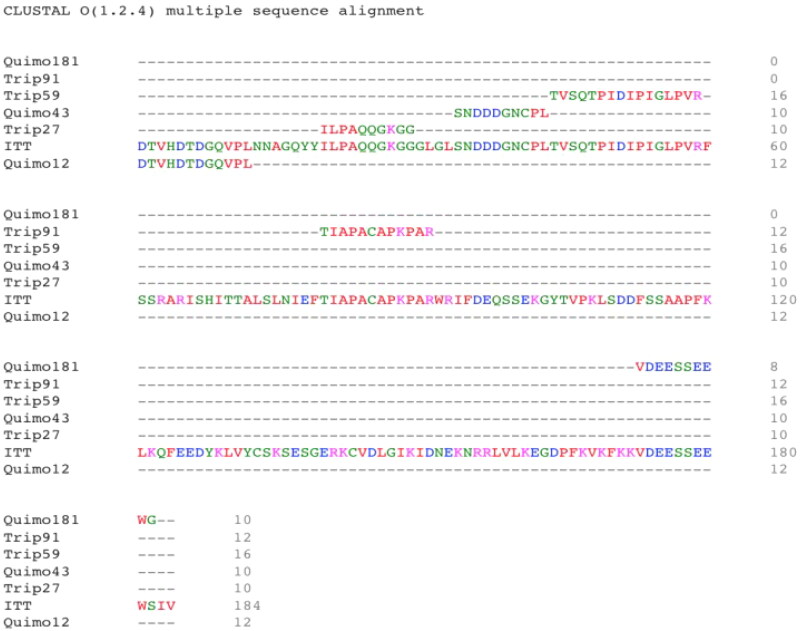
Alignment of the primary amino acid sequence of the trypsin inhibitor purified from tamarind seeds [model number 56, conformation number 287 (TTIp 56/287)] with its derivative peptides obtained by cleavage with the combined enzymes chymotrypsin and trypsin. Chymo12: peptide cleaved by chymotrypsin at position 12 when hydrolysed with a combination of trypsin and chymotrypsin enzymes; Trip27: peptide cleaved by trypsin at position 27; Chymo43: peptide cleaved by chymotrypsin at position 43; Trip59: peptide cleaved by trypsin at position 59; Trip91: peptide cleaved by trypsin at position 91 and Chymo181: peptide cleaved by chymotrypsin at position 181. Alignment performed using CLUSTAL W.

Another criterion was based on the presence of amino acids that showed more significant interaction with the lipid bilayer of bacteria with the lowest IPE, being GP, in the following position: arginine at positions: 59 and 63; Thr at positions: 70 and 71, and lysine at position: 27, according to the MD simulation, using TTIp 56/287. With these six primary peptide sequences, it was possible to align with the complete TTIp 56/287 sequence and identify the positions of amino acid residues in the respective peptide sequences (Figure 7).

After performing the alignment of the peptides, cleaved by the combined enzyme Trypsin and Chymotrypsin, the peptides were selected (Table 5) with the amino acids Arginine, Thr, and/or Lysine in their structure. The amino acids position was also used as a criterion since the peptides that have the most significant potential to interact with the membrane of GP bacteria are those that contain Arginine at positions 59 and 63; Thr at positions 70 and 71, and Lysine at position 27, according to the MD simulation using TTIp 56/287.

**Table 5.** Primary sequences of peptides generated by the ExPASy server from the *in silico* hydrolysis of the trypsin inhibitor purified from tamarind seeds [model number 56, conformation number 287 (TTIp 56/287)] with the enzymes chymotrypsin and trypsin combined with potential to interact with the membrane of Gram-positive bacteria.

Candidate peptides with the most potential for interaction with the membrane of Gram-positive bacteria					
Enzymes acting in hydrolysis	Enzyme responsible for cleavage	Cliving position	Aa sequence	Amino acids	Position^a^
Trypsin and Chymotrypsin	Chymotrypsin	12	DTVHDTDGQVPL	Threonine (T)	2 e 6
Trypsin and Chymotrypsin	Trypsin	27	ILPAQQGKGG	Lysine (K)	25
Trypsin and Chymotrypsin	Chymotrypsin	43	SNDDDGNCPL	–	–
Trypsin and Chymotrypsin	Trypsin	59	TVSQTPIDIPIGLPVR	Arginine (R)	59
	–	–	–	Threonine (T)	44 e 48
Trypsin and Chymotrypsin	Trypsin	91	TIAPACAPKPAR	Arginine (R)	91
	–	–	–	Threonine (T)	79
	–	–	–	Lysine (K)	88
Trypsin and Chymotrypsin	Chymotrypsin	181	VDEESSEEWG	–	–

^a^Position of amino acids when aligned with trypsin inhibitor purified from tamarind seeds [model number 56, conformation number 287 (TTIp 56/287)].

### 3D structure, α-helix, and structural validation

**Figure 8.**
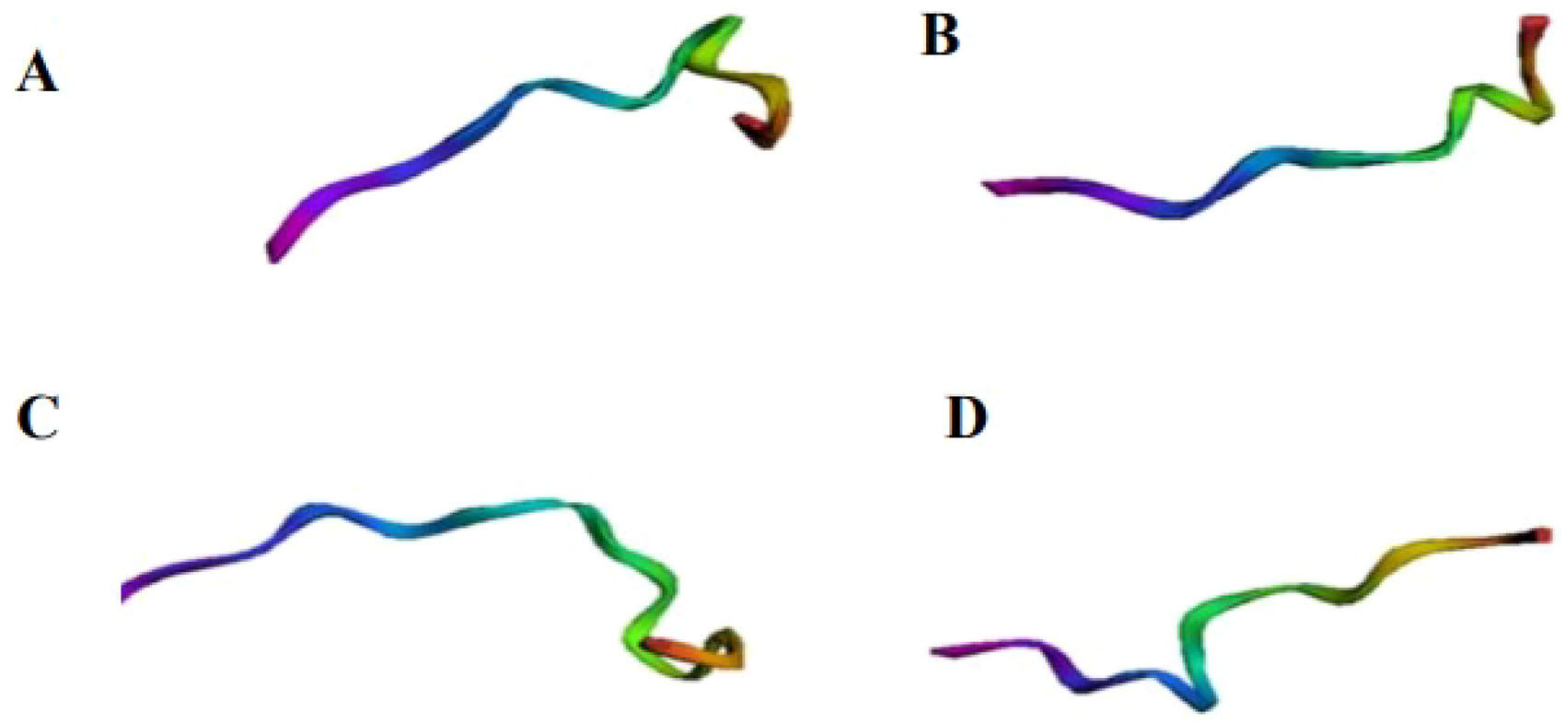
Three-dimensional structure of peptides. Generated by MolProbity from the *in silico* hydrolysis of the trypsin inhibitor purified from tamarind seeds [model number 56, conformation number 287 (TTIp 56/287)] with combined chymotrypsin and trypsin enzymes. (A) Peptide cleaved by chymotrypsin at position 12 (DTVHDTDGQVPL). (B) Trypsin-cleaved peptide at position 27 (ILPAQQGKGG). (C) Trypsin-cleaved peptide at position 59 (TVSQTPIDIPIGLPVR). (D) Trypsin-cleaved peptide at position 91 (TIAPACAPKPAR).

3D structures and theoretical validations of four peptides with antibacterial potential were generated (Figure 8 and Table 6, respectively). Among these, the peptide (Peptidotrychyme59) TVSQTPIDIPIGLPVR obtained the best results, according to the evaluated parameters, such as lower values of residues with poor bonds and angles (0.00%) and better values of favoured Ramachandran (100%) and MolProbity score (0.50), as well as having Arginine at positions 59 or 63 or Thr at positions 70 or 71 or Lysine at position 27.

**Table 6.** Validation of the three-dimensional structure of the peptides generated from the *in silico* hydrolysis of the trypsin inhibitor purified from tamarind seeds [model number 56, conformation number 287 (TTIp 56/287)] with the combined enzymes chymotrypsin and trypsin.

Peptide nomenclature	Peptide model	Ramachandran favoured (%)	Waste with bad connections (%)	Waste with bad angles (%)	Punctuation MolProbity
Peptidotrychyme12	DTVHDTDGQVPL	100	0.00	0.00	0.50
Peptidotrychyme27	ILPAQQGKGG	100	0.00	0.00	0.50
Peptidotrychyme59	TVSQTPIDIPIGLPVR	100	0.00	0.00	0.50
Peptidotrychyme91	TIAPACAPKPAR	100	0.00	0.00	0.50

**Figure 9.**
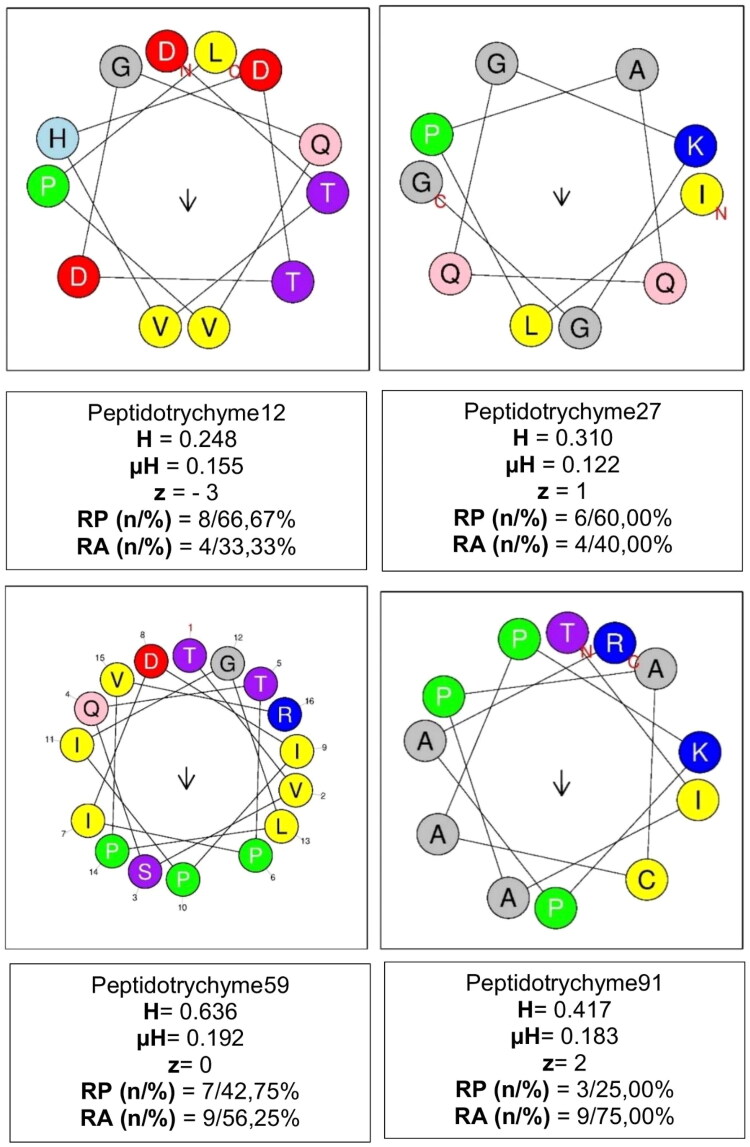
α-Helix structure of peptides. Peptidotrychyme12 – peptide 1 obtained from the hydrolysis of trypsin and chymotrypsin; Peptidotrychyme27 – peptide 2 obtained from the hydrolysis of trypsin and chymotrypsin; trypsin peptide 59 – peptide 4 obtained from the hydrolysis of trypsin and chymotrypsin; trypsin 91 peptide – peptide 5 obtained from the hydrolysis of trypsin and chymotrypsin. H: hydrophobicity; µH: hydrophobic moment; z: net charge; RP (*n*/%): polar residue in number and percentage; RA (*n*/%): nonpolar residue in number and percentage. V: nonpolar residue; T: polar residue; K: basic residue; H: histidine residue; D: acid residue; Q: glutamine residue; G nad A: glycine and alanine residue; P: proline residue. The helical structure of the peptides was obtained using the HeliQuest server. These peptides were obtained from the *in silico* hydrolysis of the trypsin inhibitor purified from tamarind seeds [model number 56, conformation number 287 (TTIp 56/287)] with the combined chymotrypsin and trypsin enzymes.

The helical structures of the four peptides with antibacterial potential were also obtained (Figure 9). Once again, the peptide (Peptidotrychyme59), with sequence TVSQTPIDIPIGLPVR, stood out with higher hydrophobicity values, hydrophobic moment, and several polar and nonpolar amino acid residues.

**Figure 10.**
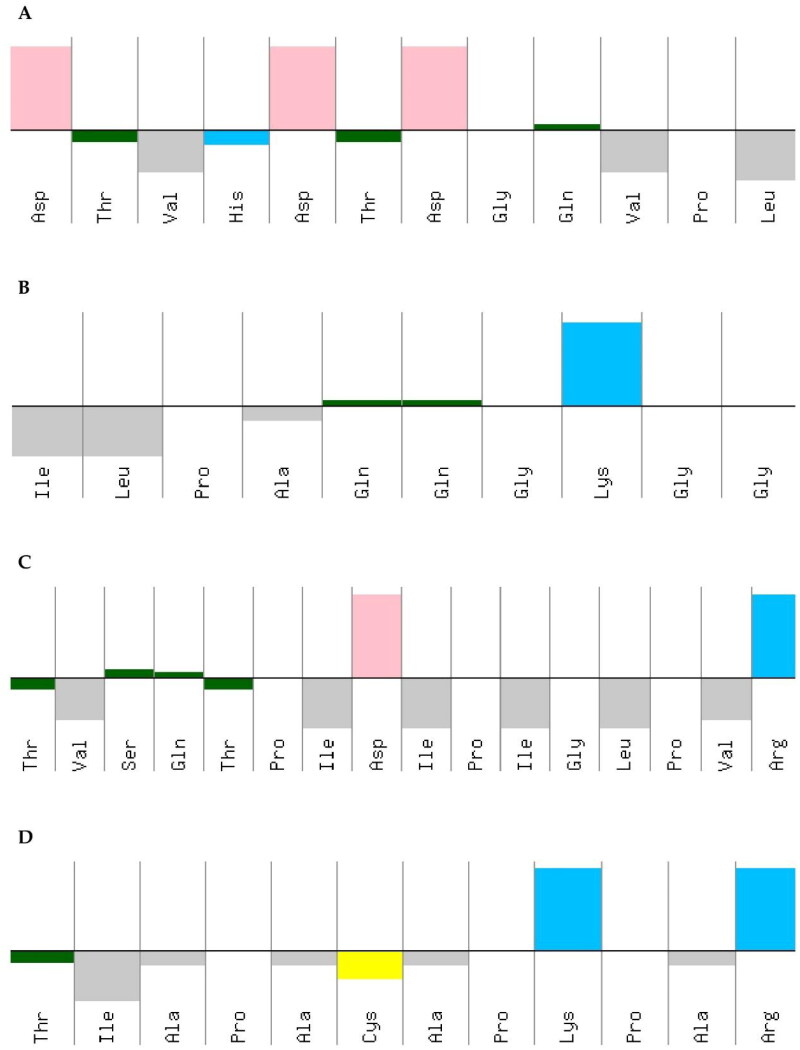
Peptide hydrophobicity characteristics. (A) Peptide cleaved by chymotrypsin at position 12 (DTVHDTDGQVPL). (B) Trypsin-cleaved peptide at position 27 (ILPAQQGKGG). (C) Trypsin-cleaved peptide at position 59 (TVSQTPIDIPIGLPVR). (D) Trypsin-cleaved peptide at position 91 (TIAPACAPKPAR).

The hydrophobic characteristics of the amino acid sequences of the peptides Peptidotrychyme12 (DTVHDTDGQVPL), Peptidotrychyme27 (ILPAQQGKGG), Peptidotrychyme59 (TVSQTPIDIPIGLPVR), and Peptidotrychyme91 (TIAPACAPKPAR) were observed (Figure 10), and Peptidotrychyme59 stood out about the amount of polar and amphipathic residues (*n* = 6).

The peptides’ hydrophobic characteristics of the amino acid sequences were obtained using the PepCalc. These peptides were obtained from the *in silico* hydrolysis of the trypsin inhibitor purified from tamarind seeds [model number 56, conformation number 287 (TTIp 56/287)] with the combined enzymes Chymotrypsin and Trypsin. The graph shows the upper part represents hydrophilicity and the lower part hydrophobicity. The colours represent the amino acid residues that make up the peptides. Yellow – cysteine residue; blue – basic residue; pink – acid residue; gray – amphipathic residue; green – polar residue; Thr – threonine; Val – valine; His – histidine; Ala – alanine; Lys – lysine; Cys – cysteine; To be – serine; Gln – glutamine; Pro – proline; Ile – isoleucine; Asp – aspartate; Gly – glycine; Leu – leucine; Arg – arginine.

However, because the TVSQTPIDIPIGLPVR (Peptidotripkyme59) presents Arginine at position 59, higher hydrophobicity, hydrophobic moment, and higher amount of polar and amphipathic residues were selected for the MD study.

### Study of MD

**Figure 11.**
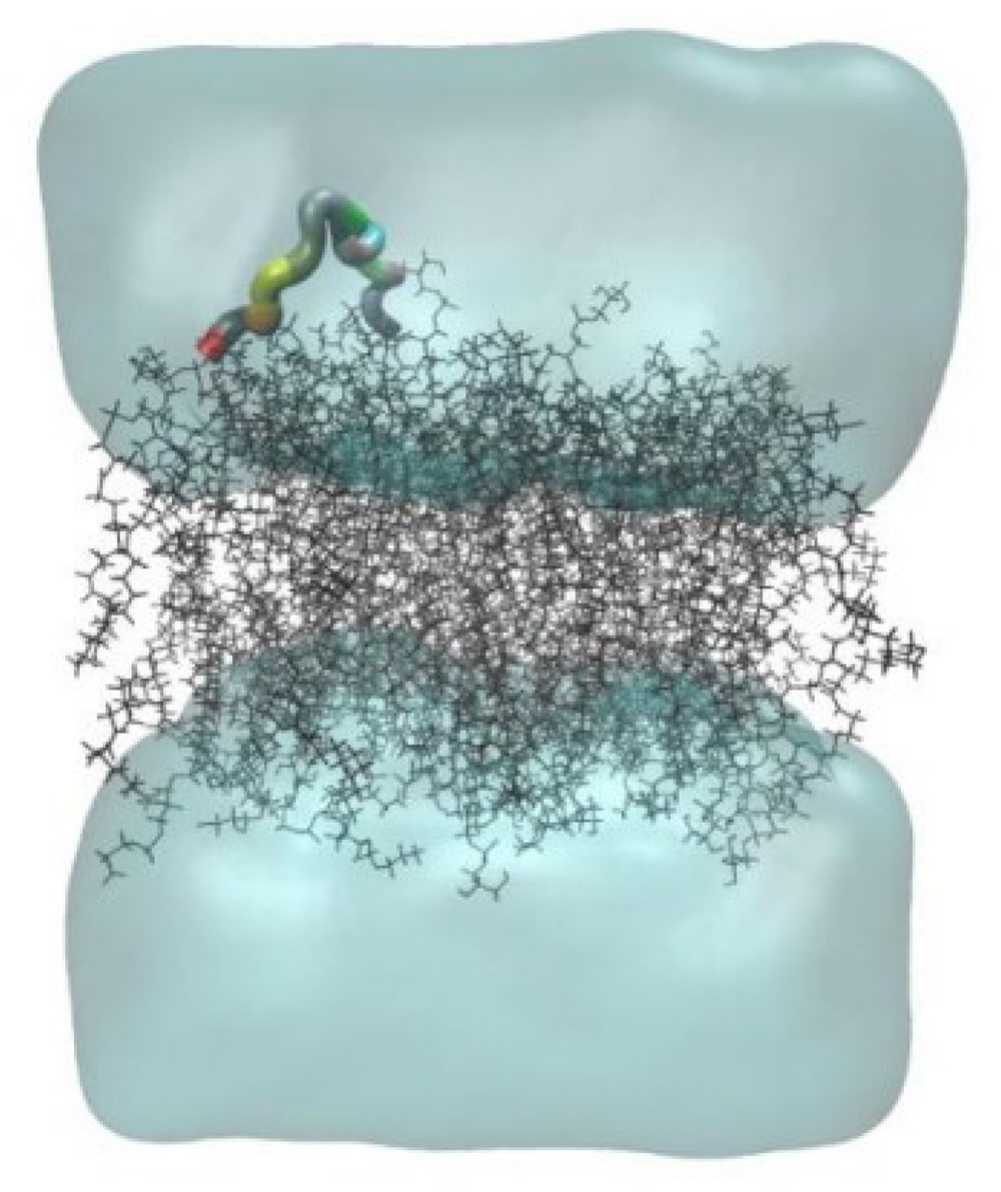
Structural behaviour of interaction between the theoretical peptide models (Peptidotrychyme59) and the membrane of Gram-positive bacteria. The peptide was obtained through hydrolysis *in silico* of inhibitor of trypsin purification of tamarind seeds [model number 56, in conformation number 287 (ITTp 56/287)] with the chymotrypsin and trypsin enzymes combined, with the bilayer model in the proportions of 3:1 of 1-palmitoyl-2-oleoilsn-glycerol-3-fosfoglycerol (POPG) to 1-palmitoyl-2-oleoyl-sn-glycerol-3-fosfoetanolamine anionic (POPE) in the representation of Gram-positive membrane. The MD was realised through GROningen MAchine for Chemical Simulations (GROMACS) *software* using version 2018.4 implemented with the Chemistry at Harvard Macromolecular Mechanics 36 (CHARMM36) force field.

The MD results showed the structural behaviour (Figure 11) and interactions of the peptide (Peptidotrychyme59) from TTIp 56/287 at the atomic level with the membranes of GP bacteria.

**Figure 12.**
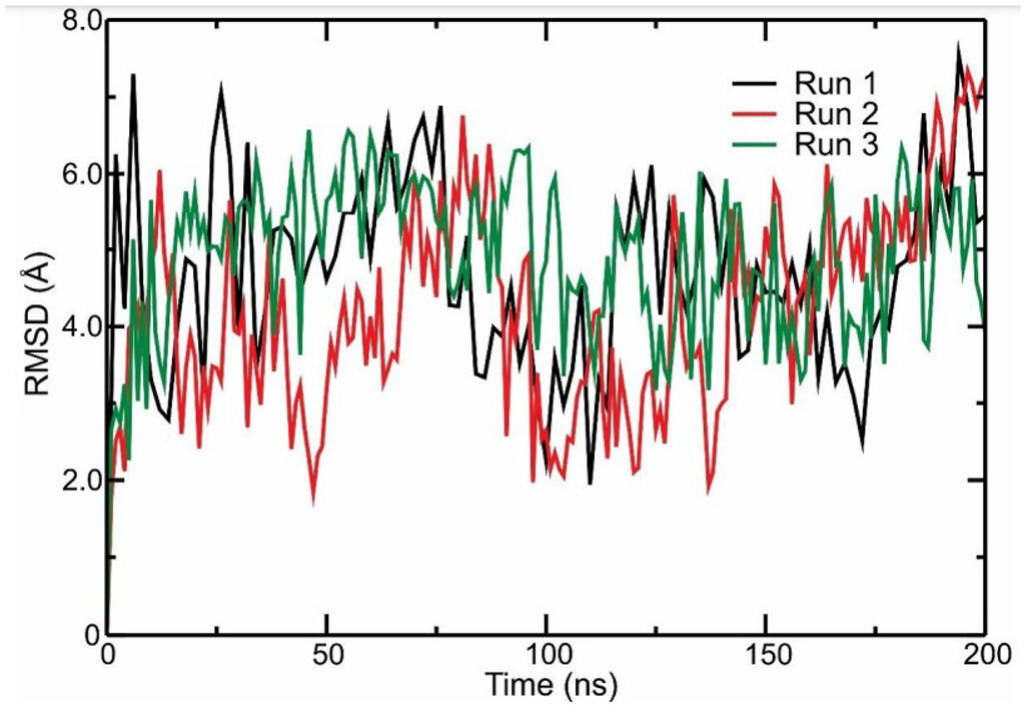
Molecular dynamics simulations of a theoretical model of peptide (Peptidotrychyme59): *Root Mean Square deviation* (RMSD) graph. The peptide was obtained through hydrolysis *in silico* of inhibitor of trypsin purification of tamarind seeds [model number 56, in conformation number 287 (ITTp 56/287)] with the chymotrypsin and trypsin enzymes combined, with the bilayer model in the proportions of 3:1 of 1-palmitoyl-2-oleoilsn-glycerol-3-fosfoglycerol (POPG) to 1-palmitoyl-2-oleoyl-sn-glycerol-3-fosfoetanolamine anionic (POPE) in the representation of Gram-positive membrane. The MD was realised through GROningen MAchine for Chemical Simulations (GROMACS) *software* using version 2018.4 implemented with the Chemistry at Harvard Macromolecular Mechanics 36 (CHARMM36) force field.

The MD simulations were performed to analyse the interval time the complex studied reached the equilibrium and the IPE between the Peptidetripquimo59 and the Gram-positive (GP) membrane. The RMSD was obtained using the C-α of the target. In the interval time of 125–200 ns, the complex analysed did not show a high variation of RMSD values. Therefore, the RMSD results suggested that the Peptidetripquimo59 showed interactions stable with the GP membrane. The IPE analysis was performed at an interval time when the complex reached equilibrium (Figure 12).

The sum of short-range Coulomb and Lennard-Jones energies realised in the IPE analysis through Gromacs software. Table 7 shows the IPE value for each replicate between peptide-GP of −518.08 kcal mol^−1^ (±204.41), −526.34 kcal mol^−1^ (±202.40), and −464.25 kcal mol^−1^ (±204.02). Therefore, the peptide analysed showed the high IPE with the GP membrane.

**Table 7.** Interaction potential energy (IPE) between the theoretical models of peptide (Peptidotrychyme59) with the Gram-positive (GP) membrane bacteria.

Interaction between peptide-GP	IPE (kcal.mol^−1^)	Standard deviation
Replicate 1	−518.08	±204.41
Replicate 2	−526.34	±202.40
Replicate 3	−464.25	±204.02

Peptidotrychyme12 – peptide 1 obtained from the hydrolysis of trypsin and chymotrypsin; Peptidotrychyme27 – peptide 2 obtained from the hydrolysis of trypsin and chymotrypsin; trypsin peptide 59 – peptide 4 obtained from the hydrolysis of trypsin and chymotrypsin; trypsin 91 peptide – peptide 5 obtained from the hydrolysis of trypsin and chymotrypsin.

**^#^**The peptide was obtained through hydrolysis *in silico* of inhibitor of trypsin purification of tamarind seeds [model number 56, in conformation number 287 (ITTp 56/287)] with the chymotrypsin and trypsin enzymes combined, with the bilayer model in the proportions of 3:1 of 1-palmitoyl-2-oleoilsn-glycerol-3-fosfoglycerol (POPG) to 1-palmitoyl-2-oleoyl-sn-glycerol-3-fosfoetanolamine anionic (POPE) in the representation of Gram-positive membrane. The MD was realised through GROningen MAchine for Chemical Simulations (GROMACS) *software* using version 2018.4 implemented with the Chemistry at Harvard Macromolecular Mechanics 36 (CHARMM36) force field.

Posteriorly, it was observed that the amino acid residues present in the peptide sequence that more interacted with the GP membrane were the Thr in position 44, followed by the Arginine (Arg) residue in position 59 due to the lowest IPE values (Table 8).

**Table 8.** Interaction potential energy (IPE) between the amino acids residues of the theoretical model of peptide (Peptidotrychyme59) with the Gram-positive (GP) membrane bacteria.

Residue	IPE (kJ.mol^−1^)
Thr	−229.85 (±100.45)
Val	−33.87 (±27.26)
Ser	−20.69 (±23.47)
Gln	−18.72 (±22.23)
Thr	−2.99 (±7.91)
Pro	−4.74 (±8.26)
Ile	−4.90 (±8.86)
Asp	−0.88 (±5.79)
Ile	−2.62 (±4.98)
Pro	−2.15 (±6.15)
Ile	−4.15 (±8.58)
Gly	−4.38 (±10.92)
Leu	−10.45 (±12.50)
Pro	−12.26 (±13.40)
Val	−4.64 (±11.75)
Arg	−160.79 (±126.57)

The peptide was obtained through hydrolysis *in silico* of inhibitor of trypsin purification of tamarind seeds [model number 56, in conformation number 287 (ITTp 56/287)] with the chymotrypsin and trypsin enzymes combined, with the bilayer model in the proportions of 3:1 of 1-palmitoyl-2-oleoilsn-glycerol-3-fosfoglycerol (POPG) to 1-palmitoyl-2-oleoyl-sn-glycerol-3-fosfoetanolamine anionic (POPE) in the representation of Gram-positive membrane. The MD was realised through GROningen MAchine for Chemical Simulations (GROMACS) *software* using version 2018.4 implemented with the Chemistry at Harvard Macromolecular Mechanics 36 (CHARMM36) force field.

Thr: threonine; Val: valine; Ser: serine; Gln: glutamine; Pro: proline; Ile: isoleucine; Asp: aspartate; Gly: glycine; Leu: leucine; Arg: arginine.

## Discussion

The search for new therapeutic agents with antibacterial action is increasing since bacterial infections have become a global concern, given the bacterial resistance to antibiotics^49^. As a result, proteins identified as PIs and their derived peptides have emerged as strong candidates in this prospection, as they naturally present protective mechanisms against pathogens^10^.

The tamarind seed trypsin inhibitor, identified as TTI, was isolated in this study. This result is demonstrated through the visualisation of SDS-PAGE of a protein band with an estimated molecular mass of approximately 20 kDa. In previous studies carried out by researchers from the NutriSBioativoS research group, the isolation of TTI had already been identified and described. In this study, once again, it was isolated as previously demonstrated by Ribeiro et al.^50^, Carvalho et al.^14^, and Costa et al.^16^ With this, it was verified that the TTI obtained has the same biochemical characteristics obtained in previous studies, confirming the reproducibility of its form of production.

Aiming to apply TTI as an antibacterial agent, evaluating this molecule’s possible cytotoxic and genotoxic effects were mandatory. Thus, in a first approach, toxicological screening studies should be performed on cell lines so that, in the future, they can be safely tested in an *in vivo* model or destined for use in humans^51^. So far, trials that evaluated cellular damage caused by TTI have not yet been performed.

Initially, in this study, it was proved that TTI, through the MTT assay, at concentrations of 0.3 and 0.6 mg.mL^−1^, did not interfere with the viability of the CHO-K1 cell line. Costa et al., in their study, evaluated the cytotoxicity of TTI; however, it was nanoencapsulated in chitosan and isolated whey protein (ECW). The nanoparticle-containing TTI, in concentrations of 0.5; 2.5, and 5.0 mg.mL^−1^, was tested in Caco-2 and CCD-18Co cell culture and also evaluated for blood toxicity at a concentration of 12.5 mg.kg^−1^ in Wistar rats, and no cytotoxic effect or toxicity were observed for this nanoencapsulated molecule^24^.

On the other hand, the genotoxicity of TTI was evaluated for the first time in this study. The data showed that TTI was not genotoxic at the highest concentration tested (0.6 mg.mL^−1^), using CHO-K1 as a cell model, as it did not cause DNA damage and cell death when compared to the negative control group. Studies evaluating the genotoxicity of trypsin inhibitors are scarce. However, considering some studies that also evaluated the potential genotoxic effects but using plant extracts with antimicrobial activity, no genotoxic effect has been observed either^52–55^.

Because of the absence of TTI genotoxicity under the tested conditions and aiming at the prospection of bioactive peptides obtained from this molecule, its digestion was performed using an *in vitro* digestion system. It was found that the TTI remained intact in the oral and gastric phases and was only digested in the intestinal phase. In the first 20 min of digestion in the intestinal phase, it is clear that TTI did not show antitrypsin activity. This data suggests that this activity was dependent on the protein structure, that is, on the natural structural conformation of the TTI. However, this particularity varies from protein to protein^56–58^.

*In vitro*, static digestion systems consist of simulating the ingestion of food and recreating the gastrointestinal tract’s physical–chemical and enzymatic environment. However, in most *in vitro* digestion protocols, the actual physiological processes, such as mixing, peristaltic movements, real-time injection of digestive enzymes, or changes in pH conditions over time, are not considered^59^.

On the other hand, this study used a universally recognised protocol that simulates *in vitro* digestion based on human digestion parameters and uses endogenous human enzymes^28^. Furthermore, *in vivo* models in humans, with proper physiological parameters, are the ideal way to simulate food digestion, but ethically and financially, it is not always feasible^60^.

Human digestion is complex, in which macronutrients ingested through food undergo mechanical and enzymatic transformation simultaneously so that hydrolytic products (peptides) are absorbed^61^. Therefore, the digestion observed in this study, even *in vitro*, indicates the susceptibility of TTI to the proteolytic enzymes, trypsin, and chymotrypsin and suggests the possibility of digestion and, consequently, the absorption of peptides obtained from TTI through specific action. This fact may also justify the systemic bioactive effects already documented for TTI (anti-inflammatory and satietogenic)^14–17^. They are probably attributed to some peptides derived from TTI, generated after digestion.

Considering these possibilities, TTI and its derived peptides, in this study, were evaluated in silico, with the perspective of revealing a new and unprecedented biopharmaceutical property, antibacterial activity. In this study, it was observed through MD that the integral molecule of TTIp 56/287 showed more significant interaction with the GP membrane, presenting IPE of −1094.97 kcal.mol^−1^, compared to the interaction with the GN, is −444,337 kcal.mol^−1^. In addition, it showed lower IPE with arginine residues at positions 63 (approximately −250 kJ mol^−1^) and 59 (approximately −150 kJ.mol^−1^), followed by Thr residues at positions 70 (approximately −50 kJ.mol^−1^), and 71 (approximately −40 kJ.mol^−1^) and Lysine at position 27 (approximately −30 kJ.mol^−1^), interacting with the GP membrane.

Considering that TTIp 56/287 showed excellent interaction activity with membranes of GP bacteria, and that it is a large molecule, composed of a total of 184 amino acid residues, the prospection of peptides with antimicrobial activity is a promising strategy.

As for the TTI-derived peptides, the theoretical cleavage of the same was initially performed by computational methods. It was useful to enable the preliminary prospection of the TTI-derived peptide with greater potential for antibacterial activity. These *in silico* strategies are efficient and low-cost to select peptides from large proteins with bioactive potential. In addition, for theoretical cleavage, the PeptideCutter of the ExPASy server used in this study included trypsin and chymotrypsin, considering the enzymatic susceptibility presented by the TTI in the *in vitro* digestion study previously showed. In addition, it provided detailed results such as cleavage site positions, peptide sequences, lengths, and molecular weights^62^.

Thus, from the theoretical model of TTI (TTIp 56/287) previously determined by de Medeiros et al.^19^, peptides with potential antibacterial activity were obtained and, among its six validated 3D structures, the peptide Peptidotrychyme59, which corresponds to residues 44–59, totalling 16 amino acid residues of the TTIp 56/287 sequence, was selected for simulation of MD with the membrane of GP bacteria. Determining the structure of a protein or peptide is essential for designating its function through possible ligands and docking sites. Peptidotrychyme59, selected for MD, presented Thr and Arginine amino acid residues at positions 44 and 59 with the lowest IPE, respectively.

Almeida et al.^63^ performed an *in silico* cleavage of the trypsin inhibitor of *Adenanthera pavonina* (ApTI) to obtain peptides with possible antimicrobial potential. They selected one, which they identified as a parent peptide. In that order, this peptide had a sequence of 18 amino acids, seven of which were compounds by Asparagine, Gly, Serine, Valine, and Proline residues. The parental peptide was submitted to *in silico* analysis, which showed a total net charge of +3 and hydrophobicity 33, and good interaction with bacterial lipid bilayers. Therefore, this peptide was synthesised to follow *in vitro* studies, and in this model, it did not show antibacterial activity at the maximum concentration tested (10 μM).

In the same study, Almeida et al.^63^ synthesised another peptide, which replaced the seven amino acid residues described above with the residues: Glutamine, Histidine, Arginine, Alanine, Phenylalanine, Lysine, and Tryptophan and named this peptide Adepamycin. Thus, in addition to the increase in a total net charge to +6, hydrophobicity 44, a well-defined amphipathic helix, and *in vitro*, they observed a bacteriostatic activity of 0.9 and 1.8 μM against *E. coli* and *S. aureus*, respectively. It is common for amphipathic cationic peptides to present an *α*-helical conformation^63^.

In addition, as in this study, they also evaluated the mechanism of interaction of Adepamycin and the representation system of the lipid bilayer composed of POPG and POPE and claimed that the amino acids Phenylalanine, Glycine, Alanine, and Histidine were the primary residues interacting with POPG phospholipids at the end of simulations^62^. Therefore, the net charge and the amphipathic structure are characteristics that allow PAM to be incorporated into the bacterial membrane, as the polar residues interact with the phosphate group and the nonpolar residues penetrate the hydrophobic core of the bilayer, compromising the cell viability of the bacteria^64^. The peptide evaluated, *in silico*, in this study also showed amphipathic residues as the ones that most interacted with the theoretical representation of the membrane of GP bacteria and, thus, included it among the peptides with antibacterial potential.

In this study, the Tripchymepeptide59 showed hydrophobicity 0.636, amphipathic helix structure, and neutral net charge at pH 7. However, Walkenhorst et al. observed in their research that the net charge of the peptide is pH dependent, where it becomes more positive at neutral pH values and less positively charged at pH 7 or higher. Furthermore, it has been shown that amphipathicity is more critical than hydrophobicity and secondary structure to promote strong binding of the peptide with antimicrobial activity (PAM) to the microbial membrane^65^.

Another study, with a peptide containing 14 amino acids pointed out as a trypsin inhibitor derived from sunflower seed 1 (SFTI-1), evaluating its antibacterial function, designed several models of hydrophobic amino acid sequence, analysing and modifying the sequences considering each of these amino acids, being Alanine, Isoleucine, Leucine, Valine, Phenylalanine, and Tryptophan. Therefore, they concluded that the peptide containing Valine, Phenylalanine, and Tryptophan showed important antibacterial activities concerning the peptides containing Alanine, Isoleucine, and Leucine, and this fact can be explained by the high correlation between the hydrophobicity of these amino acids and the antibacterial activity of PAM^66^^,^^67^.

Yang et al.^68^ evaluated a 34 amino acid peptide, Sushi 1 (S1), obtained from horseshoe crab haemocytes. Also, they performed substitutions in the amino acid sequence to improve the α-helix in peptide structures and increase the positive charge levels and the peptides’ amphipathicity. They experimented with substitution by Lysine and Arginine (SRP-1), Arginine only (SRP-2), and Lysine only (SRP-3) and observed that only SRP-2 exhibited bactericidal activity against GN and GP bacteria at 6.25 Μm. Thus, Arginine has been noted as an amino acid that demonstrates a strong electrostatic attraction with bacterial cell membranes and may facilitate the binding of peptides, increasing its antibacterial activity. In this study, Arginine composed Peptidotrychyme59, which showed excellent *in silico* interaction with the membrane of GP bacteria.

Therefore, the computational study proved crucial in the preliminary obtaining of the peptide that presented itself as a potent antibacterial candidate. Still, it is necessary to carry out *in vitro* tests with GP bacteria to prove these promising results obtained *in silico*. In addition, this preliminary prospecting points to the possibility of engineering this parental peptide (Peptidotrychyme59) to increase its antibacterial potency.

## Conclusion

The results obtained in this study suggest that TTI does not present cytotoxic activity. Furthermore, this study evaluated the genotoxicity of TTI for the first time, showing that under the tested conditions, no statistically significant nuclear cell damage was promoted, even at the highest concentration evaluated (0.6 mg.mL^−1^).

The results also showed that the *in vitro* simulated gastrointestinal digestion was efficient in hydrolysing the TTI proteins, whose peptides, in large part, were generated from the cleavage of the intestinal enzymes, trypsin, and chymotrypsin, since in the oral and gastric phases, the TTI remained intact.

The *in silico* study indicates a more significant interaction between TTIp 56/287 and the membranes of GP bacteria. Peptidotrychyme59, derived from the *in silico* cleavage of TTI, was theoretically selected as the peptide with the greatest potential for antibacterial activity. Therefore, the *in silico* findings prove a strong interaction of Peptidotrychyme59 with the theoretical representation of the membrane of GP bacteria and present it as a strong candidate for the group of antimicrobial peptides.

The result of this research may contribute to other studies, serve as a basis for *in vitro* analyses and drive future investigations with innovative products derived from TTI capable of acting on bacterial resistance.

## Supplementary Material

Supplemental Material
