# Supplementary material for: Prospecting in silico antibacterial activity of a peptide from trypsin inhibitor isolated from tamarind seed
Source: J Enzyme Inhib Med Chem. 2022 Oct 28;38(1):67–83. doi: 10.1080/14756366.2022.2134997 (PMC9621272; doi:10.1080/14756366.2022.2134997)
Supplement: Supplemental Material [file IENZ_A_2134997_SM5930.pdf]

Supplemental material

Table 1. Primary sequences and characteristics of all peptides generated by the ExPASy server from the in silico hydrolysis of the trypsin inhibitor purified from tamarind seeds [model number 56, conformation number 287 (TTIp 56/287)] with the enzymes chymotrypsin and combined trypsin.

Cleavage site position	Enzymes involved in hydrolysis	Enzyme responsible for cleavage	Resulting peptide sequence	Peptide length [aa]	Peptide mass [Da]
12	Trypsin and Chymotrypsin	Chymotrypsin	DTVHDTDGQVPL	12	1296.356
18	Trypsin and Chymotrypsin	Chymotrypsin	NNAGQY	6	665.660
19	Trypsin and Chymotrypsin	Chymotrypsin	Y	1	181.191
27	Trypsin and Chymotrypsin	Trypsin	ILPAQQGK	8	854.017
31	Trypsin and Chymotrypsin	Chymotrypsin	GGGL	4	302.330
33	Trypsin and Chymotrypsin	Chymotrypsin	GL	2	188.227
43	Trypsin and Chymotrypsin	Chymotrypsin	SNDDDGNCPL	10	1049.034
59	Trypsin and Chymotrypsin	Trypsin	TVSQTPIDIPIGLPVR	16	1706.015
60	Trypsin and Chymotrypsin	Chymotrypsin	F	1	165.192
63	Trypsin and Chymotrypsin	Trypsin	SSR	3	348.359
65	Trypsin and Chymotrypsin	Trypsin	AR	2	245.282

Information Classification: General

68	Trypsin and Chymotrypsin	Chymotrypsin	ISH	3	355.394
73	Trypsin and Chymotrypsin	Chymotrypsin	ITTAL	5	517.623
75	Trypsin and Chymotrypsin	Chymotrypsin	SL	2	218.253
79	Trypsin and Chymotrypsin	Chymotrypsin	NIEF	4	521.571
91	Trypsin and Chymotrypsin	Trypsin	TIAPACAPKPAR	12	1195.445
92	Trypsin and Chymotrypsin	Chymotrypsin	W	1	204.228
93	Trypsin and Chymotrypsin	Trypsin	R	1	174.203
95	Trypsin and Chymotrypsin	Chymotrypsin	IF	2	278.351
102	Trypsin and Chymotrypsin	Trypsin	DEQSSEK	7	821.796
104	Trypsin and Chymotrypsin	Chymotrypsin	GY	2	238.243
108	Trypsin and Chymotrypsin	Trypsin	TVPK	4	443.544
109	Trypsin and Chymotrypsin	Chymotrypsin	L	1	131.175
113	Trypsin and Chymotrypsin	Chymotrypsin	SDDF	4	482.447
119	Trypsin and Chymotrypsin	Chymotrypsin	SSAAPF	6	578.623

120	Trypsin and Chymotrypsin	Trypsin	K	1	146.189
121	Trypsin and Chymotrypsin	Chymotrypsin	L	1	131.175
122	Trypsin and Chymotrypsin	Trypsin	K	1	146.189
124	Trypsin and Chymotrypsin	Chymotrypsin	QF	2	293.323
128	Trypsin and Chymotrypsin	Chymotrypsin	EEDY	4	554.511
129	Trypsin and Chymotrypsin	Trypsin	K	1	146.189
130	Trypsin and Chymotrypsin	Chymotrypsin	L	1	131.175
132	Trypsin and Chymotrypsin	Chymotrypsin	VY	2	280.324
135	Trypsin and Chymotrypsin	Trypsin	CSK	3	336.406
141	Trypsin and Chymotrypsin	Trypsin	SESGER	6	663.642
142	Trypsin and Chymotrypsin	Trypsin	K	1	146.189
146	Trypsin and Chymotrypsin	Chymotrypsin	CVDL	4	448.535
149	Trypsin and Chymotrypsin	Trypsin	GIK	3	316.401
154	Trypsin and Chymotrypsin	Trypsin	IDNEK	5	617.657

156	Trypsin and Chymotrypsin	Trypsin	NR	2	288.307
157	Trypsin and Chymotrypsin	Trypsin	R	1	174.203
158	Trypsin and Chymotrypsin	Chymotrypsin	L	1	131.175
160	Trypsin and Chymotrypsin	Chymotrypsin	VL	2	230.307
161	Trypsin and Chymotrypsin	Trypsin	K	1	146.189
166	Trypsin and Chymotrypsin	Chymotrypsin	EGDPF	5	563.565
167	Trypsin and Chymotrypsin	Trypsin	K	1	146.189
169	Trypsin and Chymotrypsin	Trypsin	VK	2	245.322
170	Trypsin and Chymotrypsin	Chymotrypsin	F	1	165.192
171	Trypsin and Chymotrypsin	Trypsin	K	1	146.189
172	Trypsin and Chymotrypsin	Trypsin	K	1	146.189
181	Trypsin and Chymotrypsin	Chymotrypsin	VDEESSEEW	9	1109.068
184	Trypsin and Chymotrypsin	Chymotrypsin	SIV	3	317.385
		**RESULT=** 52 peptides			

Information Classification: General
